# Tracking dipeptides at work-uptake and intracellular fate in CHO culture

**DOI:** 10.1186/s13568-016-0221-0

**Published:** 2016-07-22

**Authors:** Andres Sánchez-Kopper, Max Becker, Jennifer Pfizenmaier, Christian Kessler, Andreas Karau, Ralf Takors

**Affiliations:** Institute of Biochemical Engineering, University of Stuttgart, Allmandring 31, 70569 Stuttgart, Germany; CEQIATEC, Costa Rica Institute of Technology (TEC), Cartago, Costa Rica; Evonik Nutrition & Care GmbH, Essen, Germany

**Keywords:** Dipeptide uptake, CHO cells, mAb production, Culture medium

## Abstract

Market demands for monoclonal antibodies (mAbs) are steadily increasing worldwide. As a result, production processes using Chinese hamster ovary cells (CHO) are in the focus of ongoing intensification studies for maximizing cell-specific and volumetric productivities. This includes the optimization of animal-derived component free (ADCF) cultivation media as part of good cell culture practice. Dipeptides are known to improve CHO culture performance. However, little or even conflicting assumptions exist about their putative import and functionality inside the cells. A set of well-known performance boosters and new dipeptide prospects was evaluated. The present study revealed that dipeptides are indeed imported in the cells, where they are decomposed to the amino acids building blocks. Subsequently, they are metabolized or, unexpectedly, secreted to the medium. Monoclonal antibody production boosting additives like l-alanine-l-glutamine (AQ) or glycyl-l-glutamine (GQ) can be assigned to fast or slow dipeptide uptake, respectively, thus pinpointing to the need to study dipeptide kinetics and to adjust their feeding individually for optimizing mAb production.

**Electronic supplementary material:**

The online version of this article (doi:10.1186/s13568-016-0221-0) contains supplementary material, which is available to authorized users.

## Introduction

Global biopharmaceutical markets are steadily increasing by 3–5 % per year. Markets for monoclonal antibodies (mAb), which are predominantly produced in Chinese hamster ovary cells (CHO), show even higher yearly growth rates (Walsh 2010; Aggarwal 2012, 2014). To cope with market demands, productivity of CHO-based mAb production has risen more than 100-fold since the 1990s (Birch and Racher 2006; Wurm 2004), currently reaching 2–5 g/L (and more) in 12 day-cultivations (Schaub et al. 2012). Maximum cell-specific productivities were recently published by Tabuchi and Sugiyama (2013) reporting >100 pg/cell/day.

This rise of performance data is the result of successful strain engineering in combination with process optimization. The latter mirrors all activities of process intensification using batch, fed-batch and continuous mode operations, with or without cell retention, in perfusion reactors (Jain and Kumar 2008). Certainly, medium optimization plays a decisive role for establishing best growth and production conditions in intensified bioprocesses. In essence, medium optimization should always aim at providing the best nutrient composition, maintaining reasonable prices, reproducible quality and easy access.

The ongoing search for animal-derived component free (ADCF) media aims at compositions achieving equal or even improved productivity performances compared to previous complex media (Hayashi and Sato 1976; Bottenstein and Sato 1979; Nakabayashi et al. 1982; Keen and Rapson 1995; Heidemann et al. 2000). Due to their diversity and their different boosting properties, dipeptides emerge as feasible defined medium components able to improve cell productivities. Dipeptides such as l-alanyl-l-glutamine (AQ) supplanted the use of heat-sensitive glutamine (Minamoto et al. 1991; Atanassov et al. 1998; Imamoto et al. 2010; Kim et al. 2012a, b; Imamoto et al. 2013). Glycyl-l-glutamine (GQ) additions were studied, revealing beneficial effects in murine hybridoma cell cultures (Christie and Butler 1994). l-tyrosine–containing dipeptides were also investigated as solubility of aromatic amino acids could be increased up to 250-fold in dipeptide configurations (l-tyrosyl-l-histidine (YH); Furst 1998). Recently, Kang et al. (2012) presented a comprehensive study about the industrial performance of CHO after dipeptide addition. Using l-tyrosyl-l-lysine (YK), l-tyrosyl-l-histidine (YH), l-tyrosyl-l-alanine (YA), l-tyrosyl-l-valine (YV), l-threonyl-l-phenylalanine (TF), l-histidyl-l-glycine (HG), and glycyl-l-histidine (GH), tyrosine containing dipeptides caused positive effects on culture viability and product titer. Non-tyrosine containing cultures showed variable phenotypes. Other studies by Franek and Katinger (2002) and Franek et al. (2003) reported beneficial effects of l-lysine containing peptides.

Although the history of dipeptide clearly outlines benefits for additives such as l-alanyl-l-glutamine or l-tyrosyl-X, a thorough mechanistic understanding of their intracellular functioning and fate is still missing. Fractionation studies by Christie and Butler (1994) indicated the strong impact of extracellular hydrolysis in murine hybridoma cells studying the utilization of l-alanyl-l-glutamine and glycyl-l-glutamine. On the contrary, Kang et al. (2012) recently anticipated that *…the observed fast clearance may reflect rapid transport of dipeptides into the cell rather than hydrolysis…* Apparently, the picture of dipeptides utilization, their functioning and fate inside the cells is rather unclear.

Hence, it is the motivation of this contribution to shed some light on the utilization of dipeptides by CHO cells. The l-glutamine containing additives l-alanyl-l-glutamine (AQ) and glycyl-l-glutamine (GQ) were studied as well as tyrosine containing compounds l-alanyl-l-tyrosine (AY), glycyl-l-tyrosine (GY) and l-prolinyl-l-tyrosine (PY). For comparison, other peptides were applied too: l-alanyl-l-cysteine (AC), l-alanyl-l-proline (AP), and l-prolinyl-l-cysteine (PC). Fundamental uptake kinetics were monitored for all peptides, and attention-grabbing dipeptides were selected for thorough intracellular studies. Resulting intracellular and extracellular kinetics showed that dipeptides are indeed imported into the cells and cleaved into their constitutive amino acids, which are then metabolized or secreted back to culture medium.

## Materials and methods

### Chemicals

Dipeptides were supplied by Evonik Nutrition & Care GmbH, Essen, Germany (List in Additional file 1); amino acid standards and reagents were supplied by Sigma–Aldrich (Taufkirchen, Germany). MS-grade water and MS-grade acetonitrile was purchased from Carl Roth (Essen, Germany). Amino acids and dipeptide standard stock solutions were prepared in LC–MS water and stored at 70 °C.

### Batch CHO cell cultures

The IgG1 producing strain CHO DP12#1934 (ATCC) was used for all experiments. Precultures were grown in TC42 medium (TeutoCell, Bielefeld, Germany) with 200 nM methotrexate (MTX) and 4 mM l-glutamine to a density of 0.4 × 10^6^ viable cells/mL. Aliquots were used to inoculate the main batch cultivations, again using TC42 TeutoCell medium with 200 nM MTX as basal medium. Selected single dipeptides (provided by Evonik Nutrition and Care GmbH, Darmstadt, Germany) were added to the main cultures at concentrations ranging from 4 to 6 mM. Shaking flasks (125 mL or 2 L), with working volumes of 40 and 700 mL, were used. Cultures where no dipeptide was added to the medium were performed parallel to supplemented cultures and considered as reference and named “control”. Cells were incubated at 36.5 °C, shaking at 140 rpm in 8 % CO_2_ humidified air for 8–10 days. Samples were taken daily and cell densities were measured through Trypan blue coloration on Cedex (Roche).

Characteristic cell properties were calculated to qualify the impact of dipeptide addition to culture performance. The cell specific dipeptides uptake rates *q*_*S,i*_ of *i* time intervals were estimated in batch cultures according to:

$$- \frac{1}{{\bar{c}_{X} }}\frac{{c_{S,n} - c_{S,n - 1} }}{{\Delta t_{i} }} = q_{S,i}$$

with Δ*t* as the observed time interval, $\bar{C}_{X}$*as* the average cell density of this interval, and *C*_*S,n*_ and *C*_*S,n*-*1*_ as the measured dipeptide concentrations at time points n and n − 1, respectively.

Cultivations were performed (and analyzed), at least, in duplicate.

### Sampling and sample treatment for quantification of intracellular amino acids and dipeptides

In 700 mL batch cultures, samples containing 32 × 10^6^ cells were taken. Cells were centrifuged (1300 rpm, 10 min, 4 °C) and cell pellets resuspended in 50 mL cold PBS (Phosphate buffered saline). Cells were washed twice with 5 mL PBS and cell pellets stored at −70 °C until cell extraction. Cells were extracted using the Bligh-Dyer method (Bligh and Dyer 1959) as follows: 1 mL of 1:2 CHCl_3_:MeOH was added to the cell pellet, followed by 250 µL CHCl_3_ and 250 µL water vortexed for 5 min before each addition. Phase separation was achieved by centrifugation (1300 rpm, 10 min, 4 °C). The water layer was separated, acidified with 1 % TFA (Trifluoroacetic acid), evaporated to dryness using a speed vacuum, and stored at −20 °C until analysis.

### Monoclonal antibody, amino acid and dipeptide determination

Antibody concentrations were determined by enzyme-linked immunosorbent assay (ELISA) using goat anti-human IgG F(c) as well as goat anti-human kappa chain peroxidase conjugated antibody (Rockland, USA) and SeramunBlau^®^ (Seramun, Germany) as substrate. Absorption at 450 nm was measured by Infinite^®^ 200 PRO series microplate reader (Tecan, Switzerland). Amino acids and dipeptides were analyzed simultaneously by LC–MS after dansyl chloride derivatization as described below.

### Amino acids and dipeptide dansyl chloride derivatization

Dansyl chloride derivatization was used to improve amino acids and dipeptide reverse phase chromatography retention. The derivatization protocol described by Wu et al. (2013) was used. Medium samples were diluted at 1:1500 and 50 µL borax buffer (0.1 M, pH = 9) were added to 10 µL of the diluted sample. For intracellular extracts, samples were reconstituted directly with 60 µL borax buffer. Dansyl chloride (100 µL of 20 mM; in acetonitrile) was added and the reaction was kept at room temperature for 2 h. Solutions were quenched with 100 µL 1 % formic acid and measured directly, or stored at −20 °C.

### LC-Q-TOF amino acids and dipeptide quantification

Amino acids and dipeptides were analyzed using an Agilent 1260 Infinity Bio-inert LC system coupled to and Agilent 6540 Accurate-Mass Quadrupole. Dipeptide and amino acids standards were prepared with concentrations ranging from 3 to 4000 µM, calibration ranges with correlation coefficient better than 0.98 were used. Concentrations were calculated according to a standard curve. Quantification ions are listed in the Additional file 1. Analyte identities were confirmed by matching retention times and calculated masses with mass accuracy better than 1 ppm. The LC system comprised a degasser, quaternary pump, and thermostated autosampler (maintained at 4 °C). Derivatized samples (10 µL) were injected to a reverse phase column and guard column (Aeris PEPTIDE 3.6 u XBC18 150 × 2.1 mm, Phenomenex) with a flow of 0.4 mL/min. The mobile-phase A consisted of water with 0.2 % formic acid, and B, acetonitrile with 0.2 % formic acid. The gradient was set to 17 % B at the beginning of each run, then increasing up to 80 % B in 10 min followed by a washing step and equilibration.

Mass spectrometer was configured in Extended Dynamic Range, low mass range (100–1300 m/z). Measurements were made in MS acquisition mode with acquisition rate of 2 spectra/s. JetStream electrospray ion source was configured with gas temp of 220 °C, and sheath gas temperature of 350 °C, drying gas flow 10 L/min, nebulizer set at 30 lb per square inch gauge, and sheath gas flow of 12 L/min. Capillary voltage was set to 4000 V, nozzle voltage 0 V, and fragmentor voltage to 130 V. Used reference masses were m/z 121.0509 and 922.0098 and the instrument was recalibrated every 20 samples.

Data were analyzed using Mass Hunter Workstation software (Ver.B.05.519.0, Agilent Technologies), extracting ion chromatograms of the more intense monoisotopic ion for each amino acid or dipeptide derivatives.

### Statistical analysis

The impact of dipeptide addition on maximal specific growth rates, mAB cell specific productivity and titers were statistically evaluated using a single factor ANOVA analysis against a control culture (no dipeptides in medium), considering a significance level of 95 % (α = 0.05).

## Results

### Analyzing extracellular kinetics

**Fig. 1:**
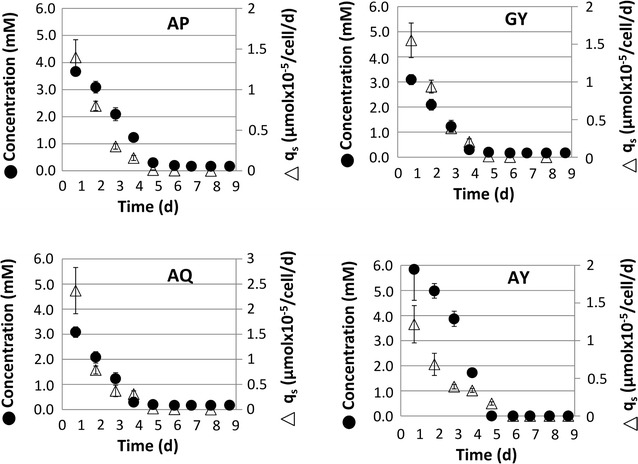
Grouped dipeptides with faster dipeptide uptake rates. Dipeptides concentration in medium (*closed circle*) decrease with a concentration dependent uptake rate (q_s_, Δ). *Error bars* describe standard deviation of biological duplicates

**Fig. 2:**
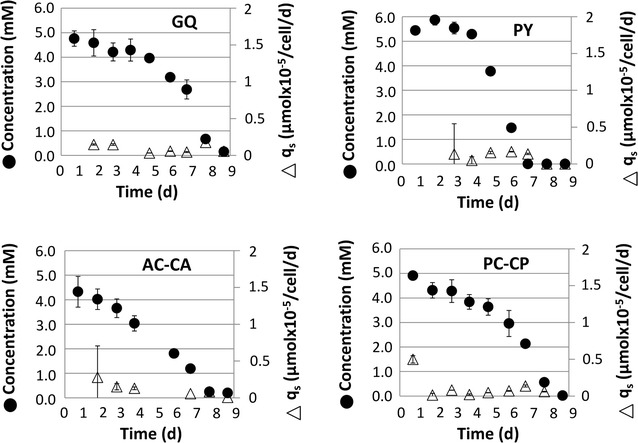
Grouped dipeptides with slower dipeptide uptake rates. Dipeptides concentration in medium (*closed circle*) decrease with slow and constant uptake rates (q_s_, Δ). *Error bars* describe standard deviation of biological duplicates

Cell culture experiments were conducted to test the performance of batch cell cultures after addition of the individual dipeptides. Taking the cell-specific dipeptide uptake rate as a criterion, two distinct phenotypes are being observed: group A, showing fast consumption, and group B, revealing slow, steady dipeptide uptake (see Figs. 1, 2).

AP, GY, AQ, and AY showed fast dipeptide uptake leading to complete dipeptide depletion after 5 days at the latest. Interestingly, this group revealed the highest daily cell-specific uptake rates of 1.5–3 µmol × 10^−5^/cells at the beginning of the batch phase. This phenotype may anticipate a diffusion-driven import with highest influx coinciding with highest concentration gradients between outer and inner conditions.

On the contrary, GQ, PY, AC-CA, and PC-CP showed much slower, but rather steady dipeptide consumption rates. As indicated, average daily depletion rates were about 0.1 µmol × 10^−5^/cells, which represent 1/10 of the values obtained for the ‘fast’ uptaked dipeptides. Apparently, uptake mechanisms for members of group B differ from those of group A.

**Fig. 3:**
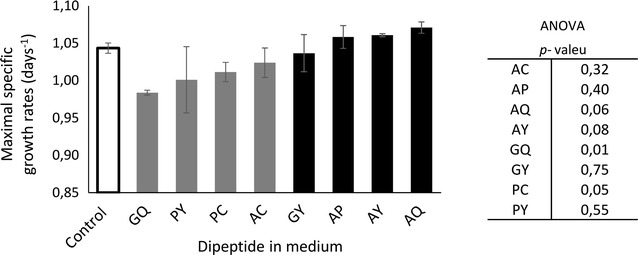
Specific growth rates for dipeptide supplemented CHO cultures compared to non-supplemented cultures (control). *Black bars* correspond to dipeptide group A showing fast consumption. *Grey bars* correspond to dipeptides group B revealing slow, steady dipeptide uptake. *Error bars* describe the deviation of duplicates

Additionally, maximum cell specific growth and mAb production rates were calculated for each supplemented batch culture and compared with a control culture (no dipeptides added). Figure 3 indicates that the addition of peptides from group B tends to slightly reduce the maximum growth rates in comparison to control, whereas dipeptides with fast uptake tend to increase growth.

**Fig. 4:**
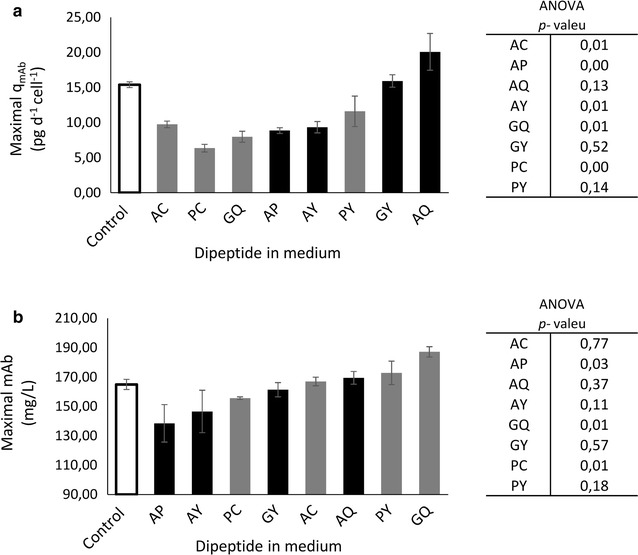
Maximal mAb production rates (**a**) and maximal mAb concentration obtained (**b**) for CHO dipeptide supplemented cultures compared to non-supplemented cultures (control). *Black bars* correspond to dipeptide group A showing fast consumption. *Grey bars* correspond to dipeptides group B revealing slow, steady dipeptide uptake. *Grey bars* correspond to dipeptides group B revealing slow, steady dipeptide uptake. *Error bars* describe the deviation of duplicates

The analysis of cell-specific mAb production reveals that only AQ outperformed, with a maximum cell-specific mAb production rate 34 % higher than the control (revealing a borderline statistical relevance). Nevertheless, the finding matches the results obtained by Imamoto et al. (2013) (Fig. 4a). The highest final concentration was obtained with GQ supplementation (+14 % total mAb production compared with the control) (Fig. 4b).

**Fig. 5:**
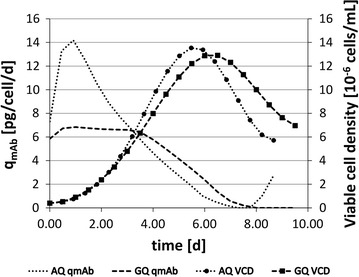
Fitted curves of specific monoclonal antibody production rates (q_mAb_) and cell growth curves (as viable cell densities, VCD) for AQ and GQ dipeptide supplemented cultures

Interestingly, the production kinetics of the best boosting dipeptides, AQ and GQ, differ significantly. Figure 5 shows the relation between viable cell numbers and cell-specific productivities. Cultures supplemented with AQ revealed maximum productivity q_mAb_ at the beginning, followed by abrupt decrease. On the contrary, GQ consumption yielded steady, but lower, q_mAb_ values, in combination with an extended period of high viable cell densities. Taken together, GQ addition achieved increased final mAb titers.

### Analyzing intracellular kinetics

Further experiments were performed to elucidate the fate of dipeptides that were consumed by the cells. AQ and AY were selected as members of group A, enabling to revisit former studies (Christie and Butler 1994; Kang et al. 2012). AC-CA served as an example of group B. It was chosen as an example of an ‘inert’ component possessing a rather bulky structure with disulfide bonds.

Batch cultivations were performed in 2 L shaking flasks (700 mL working volume) to allow sampling of relatively high cell numbers, without affecting cell growth. Samples of 32 × 10^6^ cells were taken, chilled, and three times washed (to eliminate unwanted extracellular matrix effects) before being extracted to analyze the intracellular concentrations of amino acids and dipeptides.

**Fig. 6:**
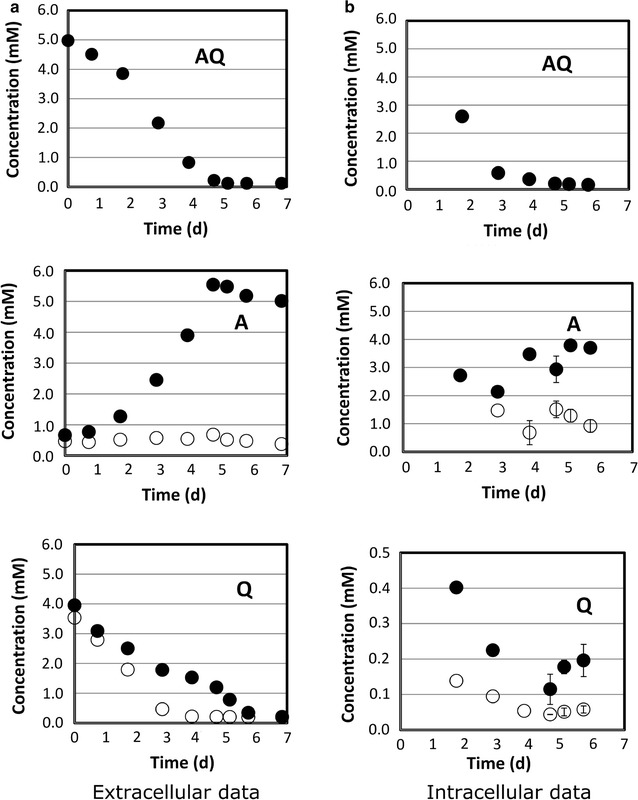
CHO culture supplemented with l-alanyl-l-glutamine (AQ). Time courses of the dipeptide and of the related amino acids are monitored in the culture medium (**a**) and in intracellular extracts (**b**). *Open circles* represent concentrations found in the control culture which did not have AQ supplementation. *Error bars* describe the standard deviation of three analytical replicates. Extracellular data from single measurements

**Fig. 7:**
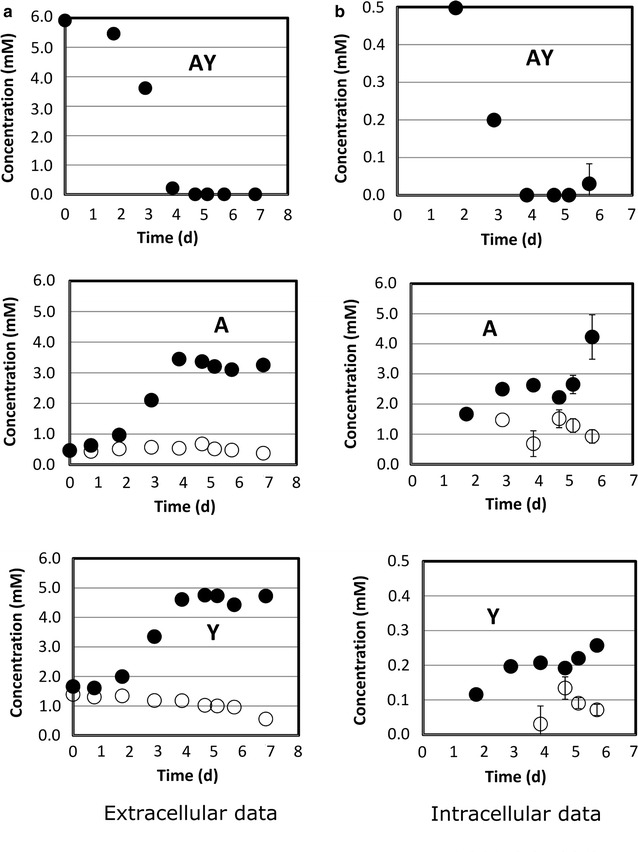
CHO culture supplemented with l-alanyl-l-tyrosine (AY). Time courses of the dipeptide and of the related amino acids are monitored in the culture medium (**a**) and in intracellular extracts (**b**). *Open circles* represent concentrations found in the control culture which did not have AY supplementation. *Error bars* describe the standard deviation of three analytical replicates. Extracellular data from single measurements

**Fig. 8:**
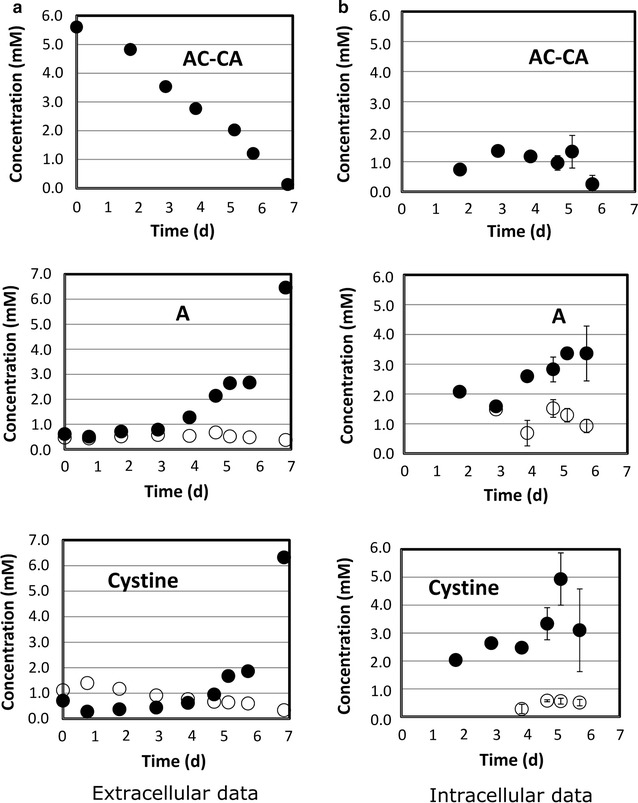
CHO culture supplemented with Alanine-cystine-alanine (AC-CA). Time courses of the dipeptide and of the related amino acids are monitored in the culture medium (**a**) and in intracellular extracts (**b**). *Open circles* represent concentrations found in the control culture which did not have AC-CA supplementation. *Error bars* describe the standard deviation of three analytical replicates. Extracellular data from single measurements

Extracellular and intracellular concentrations of selected dipeptides and their constitutive amino acids are presented in Figs. 6, 7 and 8 along with the concentrations found in the control.

Uptake kinetics of AQ, AY, and AC-CA (Figs. 6a, 7a, 8a) showed the same dynamics as already depicted in Figs. 1 and 2. Again, AQ and AY turned out to be quickly consumed and the depletion of AC-CA was much slower. Interestingly, extracellular l-alanine levels steadily increased, whereas AQ (Fig. 6a) and AY (Fig. 7a) were consumed. Glutamine was already present in the basal medium. Consequently, a steady reduction of Q titers was observed, which revealed an intermediary slow-down between 3 and 5 days. Besides, extracellular tyrosine (Y) levels increased along with the alanine courses.

These extracellular courses suggest that dipeptides were used by the cells. Glutamine (Q) appeared to be consumed internally, whereas A and Y were released, achieving almost identical amino acid levels compared to their molar fractions in the dipeptides.

To understand whether dipeptides really entered the cells or were cleaved (via extracellular proteases), intracellular measurements were performed (series B in Figs. 6, 7, 8). Indeed, AQ and AY were detected inside the cells with maximum pool sizes being measured at the beginning. Significantly, high levels of separated amino acid constituents were also observed, suggesting that dipeptides were intracellularly decomposed and their amino acids secreted individually.

The basic phenotype of AC-CA uptake was similar to AY although slower. Again, alanine (A) turned out to be taken up in AC-CA and was likewise secreted. By analogy, cysteine (measured as cystine) revealed a secretion after intake. However, intracellular levels of alanine and cystine were significantly increased compared to the control.

## Discussion

**Fig. 9:**
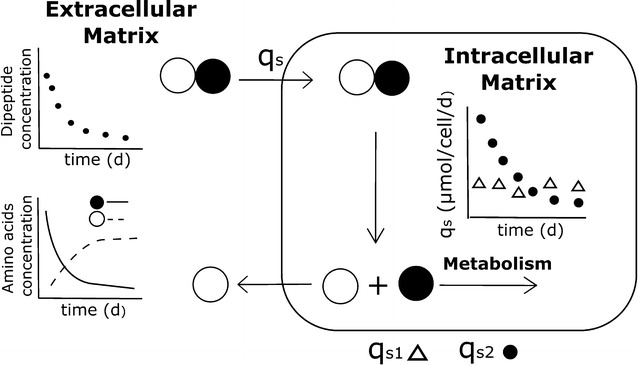
Dipeptides are transported by different mechanisms into the intracellular matrix (slow-constant uptake q_s1_ and fast-variable uptake q_s2_) where they are digested and their constitutive amino acids are either used for cell metabolisms (*closed circle*) or expelled from the cell (*open circle*)

In this study, we controlled the fate of supplemented dipeptides not only in the medium but also inside the cells. The fact that we found non-degraded dipeptides such as AQ, AY and AC-CA in the intracellular matrix (Figs. 6, 7, 8, right column B) is taken as a strong evidence that dipeptides are imported, rather than degraded by extracellular peptidase activity. Figures 1 and 2 already indicated different uptake kinetics. Further studies for AQ, AY and AC-CA confirmed the fast uptake of AQ and AY and the relatively slow uptake of AC-CA (Figs. 6, 7, 8). The observation of individual uptake kinetics suggests the existence of multiple mechanisms for their import. Once entered in the cytosol, the dipeptides are degraded to their constitutive amino acids and then selectively directed to fuel cell metabolisms (such as Q) or surprisingly exported to the culture medium (such as A). An overview of the supposed mechanism is given in Fig. 9.

For mammalian cells, two well-known dipeptide importers are described (PepT1 and PepT2). But specific dipeptide importers have not been identified for CHO-K1 cells yet (Covitz et al. 1996; Steiner et al. 1995; Daniel et al. 2006; Paulsen and Skurray 1994). However, PepT1 and PepT2 were assigned in the CHO-K1 genome as well (Hammond et al. 2012). PepT1 and PepT2 are known to show different properties with respect to uptake kinetic and selectivity (Ito et al. 2013). PepT1 is anticipated to enable fast oligopeptide import under relatively high peptide concentrations, whereas PepT2 should be active under peptide limiting conditions (Newstead 2014). From our data, two different uptake kinetics were observed. Accordingly, dipeptides were grouped in slow and fast uptake, anticipating similarities with the PepT1 and PepT2 phenotype (see Figs. 1, 2). Further efforts have to be made to identify and characterize dipeptide uptake mechanisms in CHO cells.

It was observed that dipeptides like AC-CA, PC-CP, and PY were taken up rather slowly. Interestingly, these compounds have in common rather challenging di-peptide sizes or complex ring structures in the amino acids. In this context, the slow uptake rate of GQ is surprising. Not only is glycine a small molecule, but both amino acids also have rather linear structures that should not sterically hamper their uptake. Moreover, Fig. 2 depicts that G and Q can be imported very fast as part of GY and AQ. Apparently, it is not the impact of the individual amino acids G (glycine) or Q (l-glutamine) themselves that hampers their fast uptake as GQ, but their occurrence as a dipeptide that causes the specific uptake characteristics. This observation can be taken as a hint that dipeptide uptake is very specific and that estimations for ‘similar’ compounds based on reference substances may result in poor prognosis for the distinct cases.

However, some common characteristics of dipeptide uptake can be extracted from the experiments: except for the rather bulky compound AC-CA, all other l-alanine (A) containing dipeptides were taken up very fast. This holds true for AP, AQ, and AY (see Fig. 2). Apparently, the presence of A in dipeptides eases their fast uptake (e.g. compare AQ versus GQ). This may also be true for l-tyrosine (Y), which is consumed fast in GY (compared to in GQ) and AY, but shows slow uptake properties for the relatively bulky dipeptide PY. Kang et al. (2012) also found performance improvements when Tyrosine (Y) containing dipeptides were added to CHO cultures.

As observed by our intracellular measurements, the dipeptides are imported and decomposed in their amino acid building blocks (see Fig. 9). Then the individual amino acids are either metabolized (such as Q) or secreted to the medium (such as A). Figures 6, 7 and 8 depict the common observation that fast (AQ, AY), as well as slow (AC-CA) uptake profile dipeptides cause initial increase of intracellular pools, followed by their complete disappearance during the course of cultivation. Dynamics of intracellular pool rise reflect the decline of extracellular dipeptide levels. Notably, the reduction of intracellular dipeptide pools is accompanied by the increase of intracellular levels of the related amino acids. Intracellular l-alanine (A) and l-cystine pool sizes were significantly higher than in the control experiments without dipeptide addition. On the other hand, levels of l-glutamine (Q) and l-tyrosine (Y) quickly fell down to the intracellular levels of the control cultures which had not received dipeptide supplements (see Fig. 6, 7, 8). Only an initial increase was observed that coincided with the first occurrence of the related dipeptides.

Except for l-glutamine (Q), almost equivalent levels of added dipeptides were observed as free extracellular amino acids during the course of cultivation, which indicated their relatively low metabolic demand. On the contrary, Q was metabolized immediately inside the cells. This was also true for Y, but with a lower level of metabolization.

The export of amino acids such as A or C is likely to be achieved via the ASC system, that exchanges l-alanine, l-serine and l-cysteine via a tertiary active transport mechanism from the inside towards the outside (Kyriakopoulos et al. 2013). ACS enables the refueling of intracellular, limiting amino acid pools via the export of amble ones. Trans-inhibition occurs in case of high extracellular concentrations of the designated export amino acid, whereas trans-activation is found for high driving concentration gradients between extra- and intracellular levels of the imported molecules.

As indicated in Fig. 3, the addition of group 1 dipeptides (see Fig. 1) did not impair growth rates, even showing a slight improvement for AQ. On the contrary, group 2 dipeptides slowed down the cellular growth, with GQ as the most dominant effector. Regarding final mAb titers, the resulting picture is more diverse. Although AQ addition did not exceed the control level, GQ supplementation clearly outperformed by 15 %. The reason for the benefit of GQ addition is given in Fig. 5. When comparing cell-specific mAb productivities of GQ and AQ, an early peak of productivity after AQ addition is unraveled. However, this maximum productivity could not be maintained and reduced quickly during the course of cultivation. On the contrary, GQ supplementation did not show any peak of performance, but coincided with extended periods of high cell viability. Consequently, volumetric productivities were improved after GQ addition, especially during phases of low cell growth.

For many decades, AQ and GQ have been applied as enhancers for mammalian cell cultures (Minamoto et al. 1991; Christie and Butler 1994). Indeed, both dipeptides boost cellular performance, each of them in their own way. Although GQ basically supports growth-decoupled product formation, AQ enables maximum cell-specific peaks of productivity and accelerates growth. Understanding individual dipeptide uptakes and product formation kinetics generally opens the door for an interactive process optimization. Consequently, the monitoring of dipeptide kinetics should be in the center of medium and process development in order to optimize each process considering cell-specific individuals and time-varying nutrient demands.

Furthermore, this study shows that we are only beginning to understand the impact of oligopeptide consumption on cellular physiology and to unravel underlying regulatory mechanisms. Current results do show are that dipeptides taken up, degraded inside the cells and metabolized or secreted afterwards. However, we do not know yet which regulatory regimes may be induced after dipeptide consumption and further affect cellular activities. These studies are already under way and may contribute to a knowledge-based process optimization in the future.

## Additional file

10.1186/s13568-016-0221-0 Additional material.
