# Supplementary material for: Tracking dipeptides at work-uptake and intracellular fate in CHO culture
Source: AMB Express. 2016 Jul 22;6:48. doi: 10.1186/s13568-016-0221-0 (PMC4958091; doi:10.1186/s13568-016-0221-0)
Supplement: Supplementary file 1 — 10.1186/s13568-016-0221-0 Additional material. [file 13568_2016_221_MOESM1_ESM.docx]

Additional material

Tracking Dipeptides At Work – Uptake and Intracellular Fate in CHO Culture

A. Sanchez-Kopper^1,2^, M. Becker^1^, J. Pfizenmaier^1^, C. Kessler^3^, A. Karau^3^, R. Takors^1,*^

^1^Institute of Biochemical Engineering, University of Stuttgart, Germany

^2^Costa Rica Institute of Technology (TEC), Cartago, Costa Rica.

^3^Evonik Nutrition & Care GmbH, Essen, Germany

Corresponding author:

Prof. Dr-Ing. Ralf Takors

([ralf.takors@ibvt.uni-stuttgart.de](mailto:ralf.takors@ibvt.uni-stuttgart.de)).

Institute of Biochemical Engineering, University of Stuttgart, Allmandring 31, 70569 Stuttgart, Germany

Table S1. Dipeptides used as supplements in CHO culture medium.

| Name | Formula | Abbreviations |
| --- | --- | --- |
| L-alanyl-L-glutamine | C_8_H_15_N_3_O_4_ | AQ |
| Glycyl-L-glutamine | C_7_H_13_N_3_O_4_ | GQ |
| L-alanyl-L-tyrosine | [C_12_H_16_N_2_O_4_](https://pubchem.ncbi.nlm.nih.gov/search/#collection=compounds&query_type=mf&query=C12H16N2O4&sort=mw&sort_dir=asc) | AY |
| Glycyl-L-tyrosine | C_11_H_14_N_2_O_4_ | GY |
| L-prolinyl-L-tyrosine | C_14_H_18_N_2_O_4_ | PY |
| L-alanyl-L-proline | C_8_H_14_N_2_O_3_ | AP |
| L-alanyl-L-cysteine | C_12_H_22_N_4_O_6_S_2_ | Ala-CysCys-Ala |
| L-prolinyl-L-cysteine | C_16_H_26_N_4_O_6_S_2_ | Pro-CysCys-Pro |

Table S2. Monoisotopic ions of dansyl derivatized dipeptides and amino acids used for quantification.

| Name | Abbreviations | FORMULA | Retention time (min) | Ion specie | Quantifier ion (m/z) | Qualifier ion (m/z) | Relative Abundance |
| --- | --- | --- | --- | --- | --- | --- | --- |
| dansyl-L-arginine | R-D | C18H25N5O4S | 2,299 | [M+2H]+2 | 204,589 | 205,0903 | 24,3 |
| dansyl-L-asparagine | N-D | C16H19N3O5S | 2,707 | [M+H]+ | 366,112 | 367,1149 | 18,3 |
| dansyl-L-glutamine | Q-D | C17H21N3O5S | 3,224 | [M+H]+ | 380,1275 | 381,1305 | 19,1 |
| dansyl-L-alanyl-L-cysteine | AC-1D | C24H33N5O8S3 | 3,263 | [M+H]+ | 616,1582 | 617,161 | 28,6 |
| dansyl-L-alanyl-L-glutamine | AQ-D | C20H26N4O6S | 3,582 | [M+H]+ | 451,1645 | 452,1675 | 23,3 |
| dansyl-L-serine | S-D | C15H18N2O5S | 3,89 | [M+H]+ | 339,101 | 340,1041 | 16,0 |
| dansyl-L-glutamic acid | E-D | C17H20N2O6S | 4,332 | [M+H]+ | 381,1117 | 382,115 | 18,1 |
| dansyl-L-arpartic acid | D-D | C16H18N2O6S | 4,365 | [M+H]+ | 367,096 | 368,0989 | 17,1 |
| dansyl-L-threonine | T-D | C16H20N2O5S | 4,832 | [M+H]+ | 353,1168 | 354,1191 | 21,4 |
| dansyl-glicine | G-D | C14H16N2O4S | 5,048 | [M+H]+ | 309,0904 | 310,0936 | 13,6 |
| dansyl-L-alanine | A-D | C15H18N2O4S | 5,706 | [M+H]+ | 323,1063 | 324,109 | 14,3 |
| dansyl-L-proline | P-D | C17H20N2O4S | 7,131 | [M+H]+ | 349,1219 | 350,1257 | 23,1 |
| dansyl-L-valine | V-D | C17H22N2O4S | 7,389 | [M+H]+ | 351,1374 | 352,1408 | 21,2 |
| dansyl-L-methionine | M-D | C17H22N2O4S2 | 7,447 | [M+H]+ | 383,1095 | 385,1097 | 11,2 |
| dansyl-L-tryptophane | W-D | C23H23N3O4S | 7,681 | [M+H]+ | 438,1482 | 439,1513 | 25,2 |
| dansyl-L-phenilalanine | F-D | C21H22N2O4S | 8,18 | [M+H]+ | 399,1376 | 400,1403 | 22,4 |
| dansyl-L-leucine | L-D | C18H24N2O4S | 8,297 | [M+H]+ | 365,1532 | 366,1564 | 23,3 |
| dansyl-L-isoleucine | I-D | C18H24N2O4S | 8,422 | [M+H]+ | 365,1532 | 366,1564 | 19,8 |
| di-dansyl-L-alanyl-L-cysteine | AC2-2D | C36H44N6O10S4 | 8,451 | [M+H]+ | 849,208 | 850,2105 | 41,0 |
| di-dansyl-L-cystine | C-C-2D | C30 H34 N4 O8 S4 | 8,844 | [M+2H]+2 | 354,071 | 354,5728 | 38,5 |
| di-dansyl-L-lysine | K-2D | C30H36N4O6S2 | 9,805 | [M+2H]+2 | 307,1118 | 613,2154 | 34,5 |
| di-dansyl-L-histidine | H-2D | C30H31N5O6S2 | 10,171 | [M+2H]+2 | 311,5934 | 312,095 | 36,0 |
| di-dansyl-L-cisteine | C-2D | C27H29N3O6S3 | 10,754 | [M+2H]+2 | 294,5686 | 588,1285 | 30,4 |

Table S2. Monoisotopic ions of dansyl derivatized dipeptides and amino acids used for quantification. Continued.

| Name | Abbreviations | FORMULA | Retention time (min) | Ion specie | Quantifier ion | Qualifier ion | Relative Abundance |
| --- | --- | --- | --- | --- | --- | --- | --- |
| di-dansyl-Glycyl-L-tyrosine | GY-2D | C35H36N4O8S2 | 11,221 | [M+H]+ | 705,2051 | 706,2079 | 41,2 |
| di-dansyl-L-alanyl-L-tyrosine | AY-2D | C36H38N4O8S2 | 11,329 | [M+2H]+2 | 360,1144 | 360,616 | 42,3 |
| di-dansyl-L-tyrosine | Y-2D | C33H33N3O7S2 | 11,654 | [M+2H]+2 | 324,5956 | 325,0974 | 35,2 |
